# Supplementary material for: Sex-Specific Alterations in Dopamine Metabolism in the Brain after Methamphetamine Self-Administration
Source: Int J Mol Sci. 2022 Apr 14;23(8):4353. doi: 10.3390/ijms23084353 (PMC9027322; doi:10.3390/ijms23084353)
Supplement: Supplementary file 1 [file ijms-23-04353-s001.zip › ijms-1665272-sup.pdf]

Supplementary Materials:

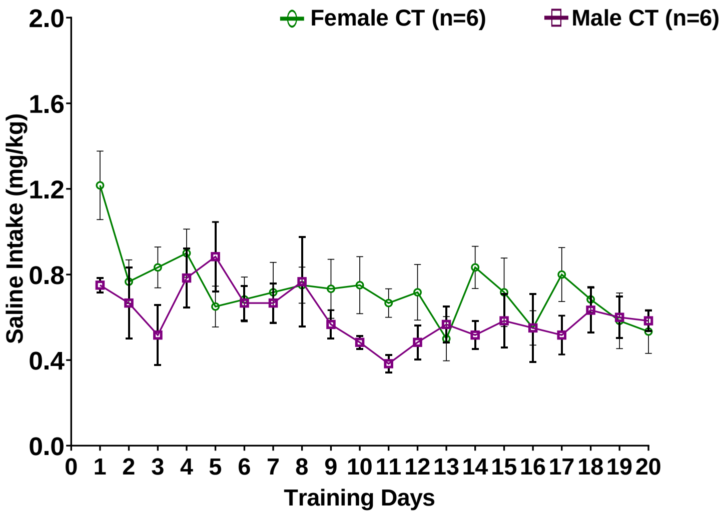

Supplementary Figure S1. Saline intake pattern of female and male rats. Two-way ANOVA comparing female and male rats showed significant effects of saline intake [F (19, 190) = 2.249, P = 0.0031], but no effect of sex [F (1, 10) = 2.804, P = 0.1250] and saline intake x sex interactions [F (19, 190) = 1.347, P = 0.1583]. Both female and male rats decreased their saline intake over time.
